# P02.170. Mindfulness-based stress reduction (MBSR) for breast cancer: a systematic review of randomized-controlled trials

**DOI:** 10.1186/1472-6882-12-S1-P226

**Published:** 2012-06-12

**Authors:** H Cramer, R Lauche, A Paul, G Dobos

**Affiliations:** 1 University of Duisburg, Complementary and Integrative Medicine, Essen, Germany

## Purpose

To assess the effectiveness of mindfulness-based stress reduction (MBSR) in patients with breast cancer.

## Methods

MEDLINE, PsychInfo, EMBASE, CAMBASE, and the Cochrane Library were screened through October 2011. Randomized controlled trials (RCTs) comparing MBSR to controls were analyzed. Risk of bias was assessed using the Cochrane risk of bias tool. For each outcome, standardized mean differences (SMD) and 95% confidence intervals (CI) were calculated, if at least 2 studies assessing this outcome were available. As a measure of heterogeneity, I^2^ was calculated.

## Results

Three RCTs with a total of 319 subjects were included. One RCT compared MBSR to usual care; one RCT compared MBSR to free choice stress management; and one three-arm RCT compared MBSR to usual care and nutrition education. MBSR was superior to usual care in decreasing depression (SMD=-0.37 [95% CI -0.65 to -0.08], p=0.01, I^2^=0%) and anxiety (SMD=-0.51 [95% CI -0.80 to -0.21], p=0.0009, I^2^=0%) but not in increasing spirituality (SMD=0.27 [95% CI -0.37 to 0.91], p=0.41, I^2^>79%).

## Conclusion

There is some evidence of effectiveness of MBSR in improving psychological health in breast cancer patients. However more RCTs are needed to underpin these results.

